# Use of the Groin Flap in Traumatic Hand Injuries

**DOI:** 10.1055/s-0045-1814122

**Published:** 2025-12-30

**Authors:** Edie Benedito Caetano, João José Sabongi Neto, Luiz Angelo Vieira, Vinicius Santos Bueno, Victor Hugo Monfrin Torres, Karen Cristina Barbosa Chaves

**Affiliations:** 1 Departament of Surgery, Faculdade de Ciências Médicas e da Saúde, Pontifícia Universidade Católica de São Paulo, Sorocaba, SP, Brazil

**Keywords:** hand injuries, iliac artery, skin graft, soft tissue injuries, surgical flaps, arteria ilíaca, enxerto de pele, lesões da mão, lesões dos tecidos moles, retalhos cirúrgicos

## Abstract

**Objective:**

To analyze the use of the groin flap in the treatment of traumatic injuries that are not very extensive, with tissue loss in the wrist and hand region.

**Methods:**

We performed 14 flaps from the inguinal region to reconstruct traumatic hand injuries. The age of the sample ranged from 21 to 56 years, and all patients were male.

**Results:**

Complete flap survival was recorded in all limbs. In seven patients, there were complications: in two, partial necrosis, for which flap debridement was necessary, and the area was covered with a skin graft. In four cases, marginal necrosis occurred, which later healed spontaneously. Partial loosening at the receiving area was observed in one case in the first week, requiring complete suturing.

**Conclusion:**

Even with the advent of microsurgical techniques, the groin flap remains a good and safe method to repair hand injuries with tissue loss, with or without loss of bone tissue.

## Resumo

**Objetivo:**

Analisar o emprego do retalho inguinal no tratamento de lesões traumáticas com perda tecidual, não muito extensas, na região do punho e mão.

**Métodos:**

Foram realizados 14 retalhos da região inguinal para a reconstrução de lesões traumáticas das mãos. A faixa etária foi de 21 a 56 anos, e todos os pacientes eram do sexo masculino.

**Resultados:**

A sobrevida completa do retalho foi registrada em todos os membros. Em sete pacientes houve complicações: em dois, necrose parcial, sendo necessário desbridamento do retalho, e a área foi revestida com enxerto de pele. Em quatro casos, ocorreu necrose marginal, que cicatrizou tardiamente de forma espontânea. A soltura parcial na área receptora foi registrada em um caso, na primeira semana, sendo necessário completar a sutura.

**Conclusão:**

Mesmo com o advento das técnicas microcirúrgicas, o retalho inguinal continua a ser um método bom e seguro de reparo de lesões com perda tecidual da mão, com ou sem perda tecido ósseo.

## Introduction

Proper coverage of complex hand injuries with tissue loss is a common yet challenging issue for hand surgeons, especially when structures such as bones, tendons, nerves, and blood vessels are exposed. In most cases, these are due to severe trauma caused by circular saws, presses, and machinery used in mechanics and agriculture, for example. Several flaps have been described to cover these injuries, 1 2 and one of them, which is used for hand injuries with soft tissue loss, is the groin flap, initially described by MacGregor and Jackson 3 in 1972, and widely used to the present day. 3 4 5 6

An ideal flap to cover injuries with tissue loss should present a low risk of surgical complications, not have a visible scar in the donor area, not present functional deficit, provide adequate quantity and quality of tissue, and have a consistent vasculo-nervous pedicle of adequate length and easy to dissect. Although this donor area has not yet been described, the groin flap offers many advantages, including reliable anatomy, minimal volume, easy adaptation to the recipient area, and minimal donor-area morbidity. In addition, it can also be used as an osteocutaneous flap by including the iliac crest. For all these reasons, the groin flap remains widely used for its simplicity, safety, and versatility. 3 4 5 6 7 8 9 10 11 12 13 A disadvantage of this procedure is the need to keep the hand close to the groin during the healing period, which lasts about 3 weeks and is very uncomfortable for the patient. In addition, because it has a short pedicle, it is difficult to use it as a free flap, and it lacks the potential for motor innervation, making it unsuitable as a functional flap. 5 6 7 8

Covering the injury site with appropriate soft tissue is essential to improve function. 1 2 5 Optimal coverage must be stable, durable, and able to withstand heavy work demands; preserve joint mobility; and have an esthetically acceptable appearance, while always prioritizing function. 5 6 7 8

The objective of the current study was to present our experience and evaluate the outcomes of the groin flap for the reconstruction of tissue losses of the hands that are not very extensive, with or without bone tissue loss.

## Materials and Methods

### Anatomy

**Fig. 1:**
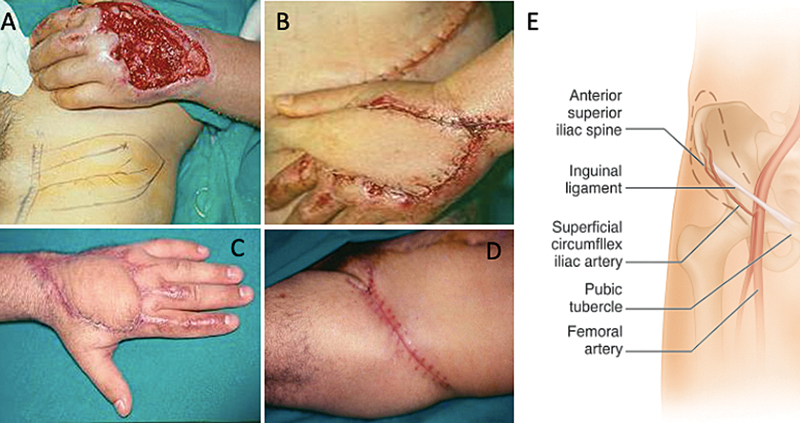
( **A** ) Flap design in the donor area; ( **B** ) flap sutured in the receiving area; ( **C** ) result in the receiving area; ( **D** ) result in the donor area; and ( **E** ) schematic drawing with the marking of the anatomical elements surrounding the flap.

The groin flap is a fasciocutaneous flap, supplied by the superficial iliac circumflex artery; the venous return is provided by the vein of the same name, which is next to the artery. The superficial iliac circumflex artery and vein originate from the femoral artery and vein, about 2 cm distally to the inguinal ligament, which joins the pubic tubercle to the anterior superior iliac spine, separating the abdomen from the lower limb. The superficial iliac circumflex artery and vein are positioned 2 cm distally to the inguinal ligament and run parallel to it, with this being the center of the flap. 3 4 5 6 7 14 Regarding the groin flap length, it can extend several centimeters beyond the anterior superior iliac spine, but the width should not exceed 10 cm. Medially, the flap should not extend beyond the femoral artery, which should be located by palpation and marked; it is safer to complete the flap dissection at a distance of 2 cm laterally from the artery ( Fig. 1 ).

### Clinical material

**Fig. 2:**
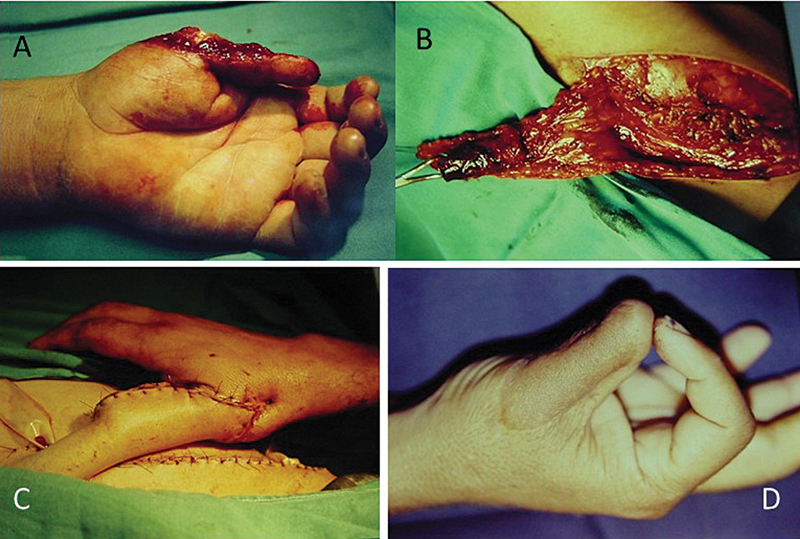
( **A** ) Osteocutaneous loss of the thumb; ( **B** ) groin flap with bone inclusion of the iliac crest; ( **C** ) flap sutured in the receiving area; and ( **D** ) final result.

A total of 14 fasciocutaneous groin flaps were performed, and, in 3, it was necessary to include a bone segment of the iliac crest ( Figs. 2 3 4 ). The flaps were used for hand and finger reconstruction between 2001 and 2017. The age of the sample ranged from 21 to 56 years, and all subjects were male. The injuries ranged in size from 5 × 8 cm to 10 × 18 cm. Surgical procedures were performed 2 to 8 days after the injury occurred. The pedicle was only disconnected from the donor area between 21 and 24 days. In most cases, the injuries resulted from workplace accidents involving presses, industrial machines, and circular saws. In two patients, injury to the extensor tendons occurred ( Figs. 5 6 ), and, in four, bone injury, including fractures and bone tissue loss ( Figs. 2 4 6 ). There was one case of tissue loss of the palmar and dorsal surfaces of the middle and annular fingers ( Fig. 7 ).

**Fig. 3:**
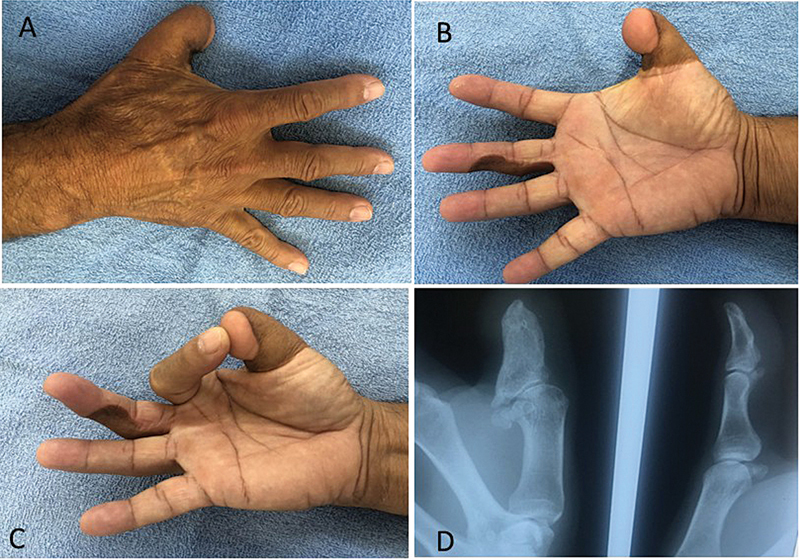
( **A** ) Reconstruction of the amputated thumb with osteocutaneous flap of the groin region; ( **B** , **C** ) neurovascular island flap from the middle finger to the thumb; And ( **D** ) radiograph of the bone flap of the reconstructed finger next to the normal thumb.

**Fig. 4:**
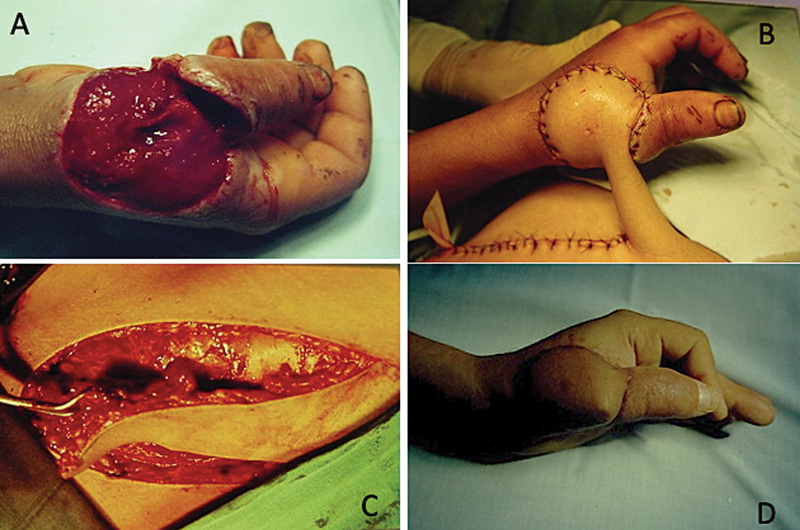
( **A** ) Osteocutaneous loss of the first metacarpal; (B) osteocutaneous flap with inclusion of the iliac crest; ( **C** ) osteocutaneous flap; and ( **D** ) final result.

**Fig. 5:**
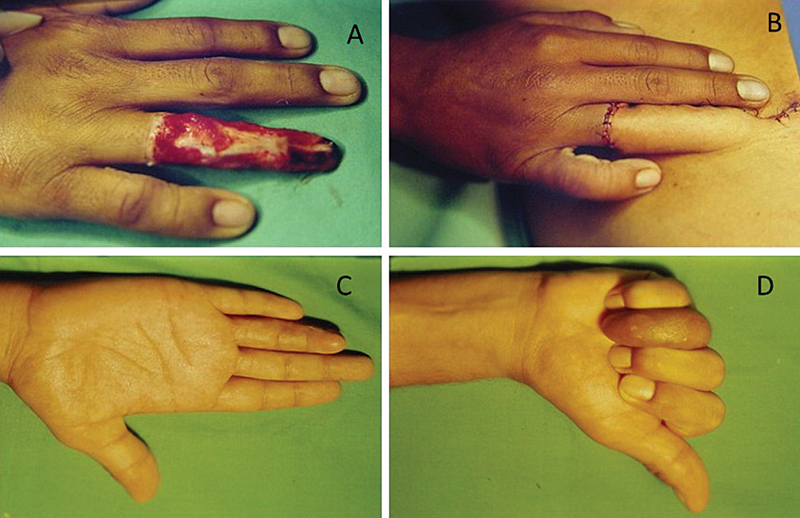
( **A** ) Dorsal ring finger injury; ( **B** ) flap from the inguinal region; ( **C** ) aspect with finger extension; and ( **D** ) aspect with finger flexion.

**Fig. 6:**
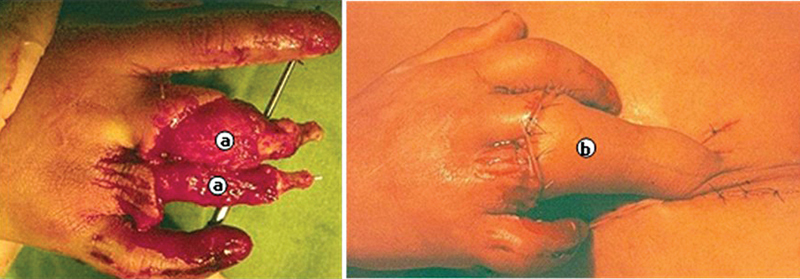
( **A** ) Injury to the middle and ring fingers with tissue loss; and ( **B** ) cover with a groin skin flap.

**Fig. 7:**
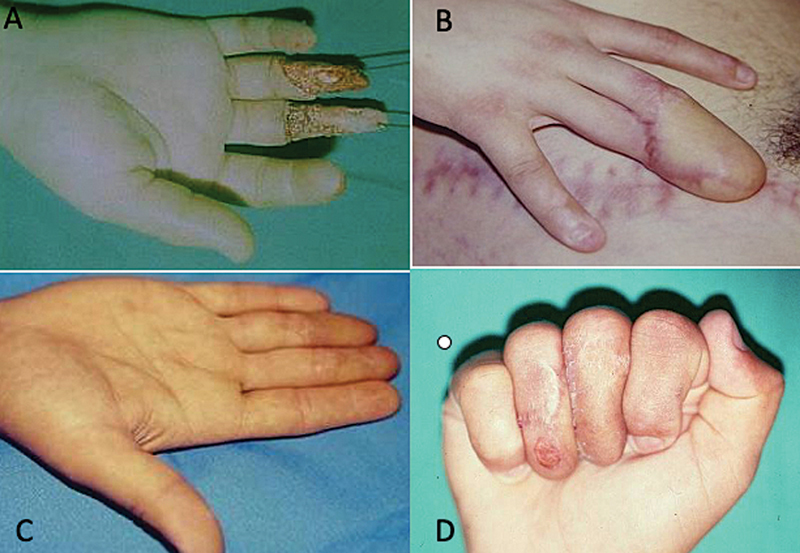
( **A** ) Tissue loss of the palmar and dorsal surfaces of the middle and annular fingers; ( **B** ) donor and recipient areas of the flap; ( **C** ) separation of the fingers in extension; and ( **D** ) aspect with digital flexion.

### Surgical procedure

The patient was positioned in dorsal decubitus. The flap was demarcated according to the needs of the receiving area. The skin, the subcutaneous tissue, and the fascia were included. In four patients with bone loss of the thumb, a segment of the iliac crest was included in the flap. The inclusion of the fascia is part of the procedure, as the vessels (superficial iliac circumflex artery and vein) are positioned on it. Dissection should be performed carefully, using a magnifying glass and electrocautery. After closing the donor area with slight tension, the procedure of covering the receiving area was completed. With a sufficiently-loose pedicle, a tube can be formed from it to leave only a minimum exposed area ( Fig. 2 ). As the flap would only be untied after 3 weeks and, during this period, the patients should keep their hand close to the groin, the procedure was performed with the limb bandaged close to the body. It was not necessary to apply an external fixator in any of the cases. All patients were discharged the day after the procedure, and they were properly instructed to keep their hand in the vicinity of the donor area.

The current study was approved by the institutional Research Ethics Committee under the number CAAE: 83985818.7.0000.5373.

## Results

**Fig. 8:**
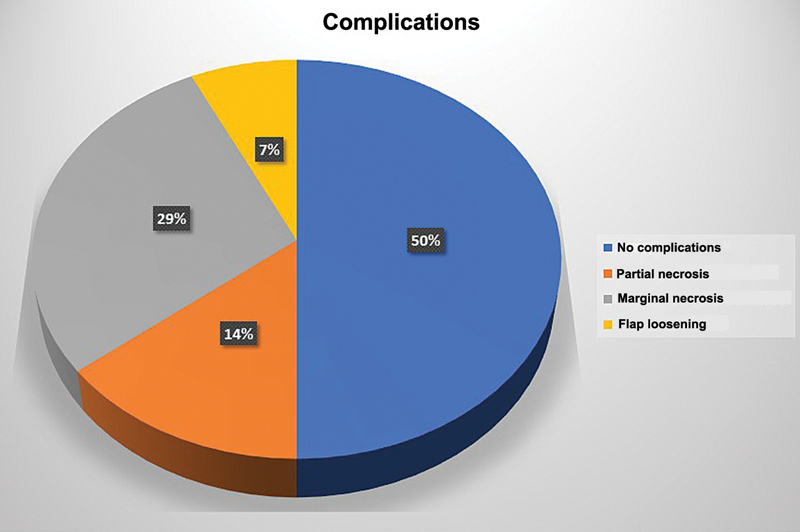
Graph with results on complications.

In 7 of the 14 limbs, no postoperative complications were observed, with primary and complete healing of the donor and recipient areas. Complete flap necrosis was not recorded in any limb. Among the seven complications were partial necrosis in two cases, which required flap debridement, and the area was covered with a skin graft; in four cases, there was marginal necrosis, which later healed spontaneously; partial loosening in the recipient area occurred in one case, in the first week, requiring completion of the suture ( Fig. 8 ) In none of the cases was the flap compromised by the patients' removal of the bandaged before the flap was unlinked.

Late treatment outcomes were assessed in 10 patients, with a mean follow-up of 24 weeks after surgery. Pain in the hand was reported by most patients, especially those involved in workplace accidents. Some patients had limitations in digital flexion or extension. Some patients also reported pain in the donor area, especially after exertion or a long walk, but none had mobility limitations in the lower limb.

## Discussion

In the current study, we present the outcomes of 14 groin flaps for the reconstruction of hand and finger injuries between 2001 and 2017. In three patients with bone loss of the thumb, a segment of the iliac crest was included in the flap. The inclusion of the fascia is part of the procedure, as the vessels (superficial iliac circumflex artery and vein) are positioned on it. The age of the sample ranged from 21 to 56 years, and all subjects were male. The injuries ranged in size from 5 × 8 cm to 10 × 18 cm. Surgical procedures were performed 2 to 8 days after the injury occurred. The pedicle was only disconnected from the donor area between 21 and 24 days in 9 of the 14 limbs. There were no postoperative complications, and the donor and recipient areas healed completely. Complete flap necrosis was not recorded in any limb. Complications occurred in seven cases: in one case, partial necrosis; in two cases, flap debridement was required, and the area was covered with a skin graft; in 4, marginal necrosis occurred, which later healed spontaneously; and partial loosening in the receiving area was observed in one case in the first week, and the suture had to be completed.

Żiluk 5 reported the outcomes of the treatment with a groin flap in 31 patients with upper-limb tissue loss, 28 male and 3 female subjects. In total, 23 patients had primary healing without problems in the donor and recipient areas. Complications were recorded in eight patients: in one, total flap necrosis occurred; in three, marginal necrosis, which required additional skin grafting after the flap was separated from the donor area; in two cases, the flaps were partially disconnected from the donor area, and a skin graft was required; and two patients had delayed healing in the donor area. The author 5 reports that the advantages of the groin flap outweigh its disadvantages, and that the outcomes of the study show that groin flaps are useful for reconstructing upper-limb soft-tissue loss.

Naala et al. 8 reported the outcomes of groin flaps in 85 patients with upper-limb soft-tissue loss, 78 male and 7 female subjects, with a mean age of 27 years. In 99% of the cases, there was good flap integration; in one patient, there was a total flap loss. The outcomes were satisfactory in most subjects, with good functional outcomes. However, complications were recorded in 22 out of the 85 patients. Marginal necrosis occurred in 10 limbs. Partial flap necrosis requiring skin graft repair occurred in 6 limbs, and local infection occurred in 4 limbs. Flap detachment requiring new surgical fixation occurred in 2 patients. According to the authors, 8 although more recent reconstruction techniques have been developed, the groin flap remains a safe and effective method for repairing upper-limb injuries with tissue loss.

Hayashi et al. 10 reported the use of the groin flap in soft-tissue losses in a reimplanted limb, with the groin flap covering the dorsal and palmar faces of the forearm. The flap healed without complications, with a satisfactory functional outcome.

Abdelrahman et al. 11 reported a modification of the groin flap method, using it without including the fascia. Anatomical studies have revealed that the superficial iliac circumflex artery is sufficient for a relatively-large segment of the flap to receive adequate blood supply without the inclusion of fascia. Naala et al. 8 presented the treatment outcomes in 77 patients with upper-limb tissue loss: the flap with fascia inclusion was used in 49 limbs, and without fascia inclusion, in 28 limbs. The treatment outcomes were satisfactory, and complications were low in both groups.

Jabaiti et al. 12 reported the outcomes of the groin flap in 34 patients, 31 male and 3 female subjects: digital injuries were the most frequent, occurring in 16 limbs. The mean injury area was of 44 (range: 12–162) cm ^2^ . Good outcomes were observed in 29 patients; however, in 1, total flap necrosis occurred; and in 4, partial flap necrosis occurred. All patients declared their satisfaction with the outcomes. The authors 12 consider that, even in the era of advanced microsurgery, the groin flap remains an excellent option for covering injuries with moderate-to-large tissue loss, and that its advantages outweigh its disadvantages.

In their first study, Molski et al. 13 reported outcomes in 97 patients with tissue defects or scar deformities in the hands, including 87 men and 10 women with a mean age of 25 years. Depending on the defect sizes, the flaps were 7 cm to 26 cm long and 4 cm to 12 cm wide. In 59 patients (61%), surgery was performed immediately after or within a few days of the lesion, while, in the remaining 38 patients, it was delayed.

Marek 15 used the osteocutaneous groin flap in eight patients to repair bone loss in metacarpals and phalanges. Flap healing was achieved in all patients, although complications, including surgical wound infection, partial flap necrosis, and an inflammatory reaction at the suture sites, were observed in 7 limbs. The author 15 concluded that the osteocutaneous groin flap is a good solution for associated bone and soft-tissue losses.

Gupta et al. 16 presented the outcomes of the groin flap in 25 children aged 9 years or older who suffered hand tissue loss as a result of electrical burns. In most cases, the losses involved exposure of tendons and bones. Flap healing was achieved in all cases, including two cases of partial necrosis requiring skin graft coverage. Suture dehiscence was also observed in the donor area in two children. Tests performed on average 1 year after surgery showed good functional outcomes. The children were well, able to play and perform their daily activities normally.

Devarasetty et al. 17 conducted a study involving 88 pedicled groin flaps used to cover upper-limb injuries. The patients had a median age of 35 years and underwent a mean of 4 surgeries throughout the treatment. The most commonly observed complication was finger stiffness. Other common complications were partial flap loss (in 38% of the cases) and infection (in 32%). This study reported a higher partial flap loss rate than that of our sample, which showed only 14%. High-energy injuries are more likely to require more procedures during treatment. The analysis revealed no significant differences in wound complications across patient or injury characteristics.

## Conclusion

The treatment outcomes of injuries to the hands that are not very extensive, with or without bone tissue loss, using the groin flap, were satisfactory. Even with the advent of microsurgical techniques, the groin flap remains a good and safe method for repairing hand injuries with tissue loss, with or without bone loss.
